# Genome-wide identification and characterization of the *ZmBED* gene family in maize and its putative roles in drought adaptation via rhizosheath formation

**DOI:** 10.3389/fpls.2026.1884999

**Published:** 2026-07-07

**Authors:** Shi-Kai Cao, Huijuan Zhao, Tian Ma, Zizhen Fu, Ze Feng, Rui Liu, Hewei Du, Jianhua Zhang

**Affiliations:** 1 School of Life Science, Yangtze University, Jingzhou, China; 2 Hubei Key Laboratory of Waterlogging Disaster and Agricultural Use of Wetland Yangtze University, Jingzhou, China; 3 Department of Biology, Hong Kong Baptist University, Hong Kong, China

**Keywords:** abiotic stress response, drought stress, gene expression, maize, phylogenetic analysis, rhizosheath, Zf-BED family

## Abstract

The Zf-BED (zinc finger-BED) domain-containing family comprises transcription factors involved in plant growth and stress responses; however, its functional landscape in maize (*Zea mays*) remains largely unexplored. In this study, we performed a genome-wide identification and characterization of the *ZmBED* gene family in maize. A comprehensive analysis of 25 ZmBED proteins was conducted, including assessment of physicochemical properties, phylogenetic reconstruction, synteny and *cis*-regulatory element analyses, as well as expression profiling under multiple abiotic stresses. Our results show that *ZmBED* genes are unevenly distributed across maize chromosomes and exhibit conserved domain architectures but diverse gene structures. Expression analysis revealed tissue-specific patterns and distinct transcriptional responses to cold, heat, salt, UV, and drought stress. Notably, four *ZmBED* genes (*ZmBED7*, *16*, *19*, and *20*) were significantly upregulated in root tissues under drought stress, coinciding with a pronounced increase in rhizosheath mass. Collectively, these findings provide a systematic characterization of the ZmBED family and identify four candidate members whose expression is associated with drought adaptation and rhizosheath formation in maize, providing a foundation for future functional studies.

## Introduction

Maize (*Zea mays*) is a cornerstone crop for global food security, yet its yield and quality are frequently threatened by multiple abiotic stresses, including drought, high salinity, extreme temperatures, reactive oxygen species (ROS) accumulation, and heavy metal toxicity (Salika and Riffat, 2021). Deciphering the molecular mechanisms underlying maize stress responses and identifying pivotal regulatory genes have therefore become central priorities in modern breeding research.

Transcription factors (TFs) serve as master regulators of stress adaptation by recognizing specific *cis*-acting elements in target gene promoters, thereby activating or repressing stress-responsive gene expression (Schwechheimer and Bevan, 1998). In maize, major TF families implicated in abiotic stress tolerance include NAC, AP2/ERF, bHLH, bZIP, MYB, WRKY, and zinc finger proteins, each with well-documented roles (Park et al., 2025). For instance, NAC members ZmSNAC1 and ZmSNAC06 promote root development and drought resistance (Wang et al., 2025; Shi et al., 2026). AP2/ERF factors ZmDREB1A and ZmRAV1 modulate inositol metabolism and antioxidant defense (Li et al., 2025b; Liu et al., 2025). Other families like bHLH, bZIP, MYB, and WRKY regulate ABA signaling, stomatal closure, and ROS scavenging (Ma et al., 2018; Li et al., 2019; Li et al., 2025a; Li et al., 2026). Despite these advances, one important but relatively understudied TF class in maize is the zinc finger protein family.

Zinc finger proteins are structurally conserved DNA-binding proteins classified into nine subfamilies (C2H2, C3H, C3HC4, etc.) based on cysteine/histidine coordination (Berg and Shi, 1996). Among these, the C2H2 subfamily is the most abundant and is further divided into plant-specific Q-type (containing the QALGGH motif) and C-type (lacking this motif) (Kubo et al., 1998; Lu et al., 2024). Within the C2H2 subfamily, BED-type zinc finger proteins (Zf-BED) are distinguished by a conserved BED domain capable of binding DNA, RNA, or proteins (Babu et al., 2006). In animals, Zf-BED proteins regulate key processes such as IGF2 repression (ZBED6) and Wnt signaling (ZBED3) (Ali et al., 2015; Gu et al., 2024). In plants, limited reports indicate that the rice Xa1 protein uses its BED domain for pathogen recognition (Yoshihisa et al., 2025), oat Pc94 confers crown rust resistance (Moreau et al., 2026), and a rice *ZBED* gene enhances both disease resistance and drought tolerance (Zuluaga et al., 2020). Moreover, the Zf-BED domain of the snapdragon *Tam3* transposase controls nuclear import and transposition activity under low temperature (Zhou et al., 2017). However, in maize—a crop where abiotic stress tolerance is of paramount importance—research on Zf-BED family members remains nascent, and a systematic identification and functional characterization is still lacking.

Additionally, gramineous plants can develop rhizosheath structures (soil particles bound by root exudates) that enhance tolerance to drought, low phosphorus, and high salinity (R. W. Duell and Peacock, 1985; Steiner et al., 2024), suggesting that root-related adaptive mechanisms may involve novel regulatory components. Against this background, the present study systematically identifies Zf-BED family members in the maize genome using bioinformatics approaches, and analyzes their evolutionary relationships, structural features, and potential functional characteristics. This work aims to establish a foundation for future mechanistic investigations into the roles of Zf-BED proteins in maize stress responses and rhizosheath formation.

## Materials and methods

### Plant growth and drought treatment

Maize (*Zea mays* B73) seeds were planted in plastic pots (10 cm × 8.5 cm) filled with air-dried fluvo-aquic silt loam (pH 7.1) from the experimental farm of Yangtze University, Jingzhou, sieved through a 10-mm mesh. At the three-leaf stage, drought stress was imposed at 50% field capacity for 14 days by daily pot weighing and watering every 2~3 days according to evapotranspiration loss; control plants were maintained at 70~75% field capacity. Plants were grown under natural diurnal conditions (28/20 °C day/night, 3600~4000 lux at canopy height). On days 0 and 14, rhizosheath fresh weight, primary root length, and primary root tips (≈ 0.2 g) were collected, frozen in liquid nitrogen, and stored at –80 °C. Three biological replicates were used per treatment per time point.

Tobacco (Nicotiana tabacum) plants, obtained from laboratory-maintained stock, were grown at 21 °C under a 12 h light/12 h dark photoperiod.

### Identification of ZmBED family members in the maize genome

The maize genome sequence and its corresponding annotation files were downloaded from the MaizeGDB database (https://maizegdb.org/). Protein sequences of rice genes previously reported to contain the Zf-BED domain were retrieved from NCBI (https://www.ncbi.nlm.nih.gov/). These rice sequences were used as queries to identify candidate Zf-BED family members in the maize genome using the BLAST function implemented in TBtools (Chen et al., 2020; Chen et al., 2023). To validate the candidate members, all retrieved maize protein sequences were subjected to domain verification using the online tools InterPro (https://www.ebi.ac.uk/interpro/) and NCBI CD-Search (https://www.ncbi.nlm.nih.gov/Structure/bwrpsb/bwrpsb.cgi). Sequences lacking a complete Zf-BED domain or entirely missing the domain were excluded from further analysis.

### Physicochemical property analysis

The physicochemical properties of the maize ZmBED family members were analyzed using the ProtParam tool on the ExPASy server (https://www.expasy.org/). For each protein sequence, the following parameters were calculated: number of amino acids, molecular weight (kDa), theoretical isoelectric point (pI), instability index, and grand average of hydropathicity (GRAVY). All analyses were conducted using default parameters.

### Subcellular localization of ZmBED proteins

Subcellular localization of ZmBED proteins was predicted using WoLF PSORT, Plant-mPLoc, and BUSCA. A consensus localization was assigned based on agreement between at least two tools, with Plant-mPLoc prioritized in the event of complete disagreement. For experimental validation, *ZmBED11*, *18*, and *21* were amplified and recombined into the pSuper1300-GFP vector using the Vazyme ClonExpress^®^ II Kit. The recombinant plasmids were transformed into *Agrobacterium tumefaciens* EHA105. The resulting *Agrobacterium* cultures were infiltrated into tobacco leaves, and GFP fluorescence was observed under a laser scanning confocal microscope as described (Cao et al., 2022). Primers are listed in **Supplementary Table 1**.

### Conserved motif, domain analysis and gene structure analysis of ZmBED family members

To characterize the structural features of ZmBED family members, conserved motifs were predicted from the protein sequences using the MEME online tool (https://meme-suite.org/meme/tools/meme). Protein domain information was retrieved using NCBI CD-Search (https://www.ncbi.nlm.nih.gov/Structure/bwrpsb/bwrpsb.cgi) and InterPro (https://www.ebi.ac.uk/interpro/). Based on the obtained motif and domain annotations, gene structure analysis was conducted. Diagrams illustrating the conserved motifs and domains were generated using the “Domain & Motif Plot” function in TBtools (version 1.098) with default parameters (Chen et al., 2020; Chen et al., 2023).

### Chromosomal distribution of ZmBED family members

Chromosomal localization information for the ZmBED family members was extracted from the maize B73 reference genome annotation file (RefGen_v4). A physical map illustrating the chromosomal distribution of these genes was then generated using the “Gene Location Visualize from GFF” function in TBtools (version 1.098) with default parameters (Chen et al., 2020; Chen et al., 2023).

### Promoter *cis*-regulatory element analysis

The 2, 000 bp promoter sequences immediately upstream of the start codon of each *ZmBED* gene were extracted from the maize B73 genome (RefGen_v4) using TBtools. These sequences were then submitted to the PlantCARE database (https://bioinformatics.psb.ugent.be/webtools/plantcare/html/) for *cis*-regulatory element prediction using default parameters. The predicted elements were subsequently visualized with TBtools (Chen et al., 2020; Chen et al., 2023).

### Phylogenetic tree construction

Genome and annotation files for *Arabidopsis thaliana* and rice (*Oryza sativa*) were downloaded from the Ensembl Plants database (https://plants.ensembl.org/index.html). To identify Zf-BED family members in Arabidopsis and rice, BLAST analysis was performed using the protein sequences of the previously identified *ZmBED* genes as queries against the protein sequences of both species. A phylogenetic tree was then constructed using the “One Step Build a ML Tree” function in TBtools with default parameters (Chen et al., 2020; Chen et al., 2023). The resulting tree was subsequently visualized and refined using the online tool iTOL (https://itol.embl.de/).

### Prediction of the protein-protein interaction network

The protein-protein interaction (PPI) network of maize ZmBED proteins was retrieved from the STRING database (https://cn.string-db.org/) (Chen et al., 2021b). The obtained network data were then imported into Cytoscape (version 3.10.0) for visualization and refinement. A finalized interaction network of ZmBED proteins was subsequently generated.

### Expression pattern analysis under stress conditions

Transcriptome data of *ZmBED* members in maize, including expression profiles across different tissues and under abiotic stress conditions, were retrieved from the qTeller database (https://qteller.maizegdb.org/genes_by_name_B73v4.php). The retrieved data were then normalized and visualized as a heatmap using TBtools software (Chen et al., 2020; Chen et al., 2023).

### RT-qPCR analysis

Total RNA was extracted from the roots of control and drought-treated maize seedlings using the FastPure Plant Total RNA Isolation Kit (Vazyme). Reverse transcription was performed using 1 μg of RNA with the PrimeScript RT Kit containing gDNA Eraser (Takara). RT-qPCR was carried out on a CFX96 system (Bio-Rad) using SYBR Green master mix (Vazyme). The maize *ZmActin* (*GRMZM2G126010*) gene was used as an internal control. Three biological replicates and three technical replicates were analyzed for each sample. Relative expression levels were calculated using the 2^-ΔΔCt^ method, with the control group serving as the calibrator (Song et al., 2023). All qPCR primers used in this study are listed in **Supplementary Table 1**.

### Collinearity analysis

Genome annotation and sequence files for maize, *Arabidopsis thaliana*, and rice (*Oryza sativa*) were downloaded from MaizeGDB and Ensembl Plants, respectively. Genome-wide comparative analysis was performed using the “One Step MCScanX” tool in TBtools with BLASTP (E-value ≤ 1e-5), which generated intra-species collinearity for maize as well as inter-species collinearity between maize and Arabidopsis and between maize and rice. The resulting collinearity relationships were visualized using the “Advanced Circos” tool for intra-species comparisons and the “Multiple Synteny Plot” tool for inter-species comparisons in TBtools (Chen et al., 2020; Chen et al., 2023).

## Results

### Identification and physicochemical properties of maize Zf-BED family members

A total of 25 Zf-BED family members were identified in the maize genome and were designated *ZmBED1* to *ZmBED25* (**Table 1**). Physicochemical property analysis showed that the length of the amino acid residues ranged from 135 to 1, 375, corresponding to molecular weights ranging from 15, 475.69 Da to 154, 303.09 Da. The theoretical isoelectric points (pI) varied from 5.22 to 9.38, with 14 proteins classified as acidic (pI < 7) and 11 as basic (pI > 7). The instability index ranged from 40.67 to 59.49, all above 40, which computationally indicates that these Zf-BED domain-containing proteins are likely to be unstable and potentially prone to degradation *in vitro*. The grand average of hydropathicity (GRAVY) values was negative for all members, suggesting that all ZmBED proteins are hydrophilic.

**Table 1:** Physicochemical properties and subcellular localization prediction of ZmBED family members.

Gene ID	Protein name	Subcellular localization	Protein length (aa)	Theoretical pI	Molecular mass (Da)	Instability index	GRAVY
*Zm00001d003128*	ZmBED1	Nucleus	644	6.69	73617.31	51.38	-0.312
*Zm00001d003194*	ZmBED2	Nucleus	439	7.54	50136.36	51.79	-0.364
*Zm00001d006692*	ZmBED3	Nucleus	397	8.46	45158.48	40.67	-0.687
*Zm00001d008443*	ZmBED4	Nucleus	696	6.46	79120.29	47.90	-0.323
*Zm00001d010619*	ZmBED5	Nucleus	487	8.58	54655.32	49.76	-0.375
*Zm00001d010895*	ZmBED6	Nucleus	409	5.85	47865.26	41.69	-0.585
*Zm00001d011158*	ZmBED7	Nucleus	1154	6.65	130827.84	53.30	-0.737
*Zm00001d013336*	ZmBED8	Nucleus/ chloroplast	1375	5.56	154303.09	43.65	-0.278
*Zm00001d015283*	ZmBED9	Nucleus	354	8.28	39913.15	46.33	-0.153
*Zm00001d017846*	ZmBED10	chloroplast	616	9.38	69432.05	59.49	-0.581
*Zm00001d022534*	ZmBED11	Nucleus	390	6.43	43639.45	47.00	-0.292
*Zm00001d023717*	ZmBED12	Nucleus	495	6.58	55919.82	47.24	-0.335
*Zm00001d024229*	ZmBED13	Nucleus	798	5.96	90999.16	42.14	-0.386
*Zm00001d024275*	ZmBED14	Nucleus	682	7.25	77777.32	49.36	-0.444
*Zm00001d025207*	ZmBED15	chloroplast	278	8.86	31407.72	47.41	-0.551
*Zm00001d026358*	ZmBED16	Nucleus	600	6.28	68735.20	54.86	-0.387
*Zm00001d028972*	ZmBED17	Nucleus	212	5.35	24109.43	51.85	-0.413
*Zm00001d033903*	ZmBED18	Nucleus	481	6.29	53825.44	46.94	-0.245
*Zm00001d034670*	ZmBED19	Nucleus	811	6.11	90580.00	43.69	-0.343
*Zm00001d039328*	ZmBED20	Nucleus	741	7.16	82686.84	43.16	-0.355
*Zm00001d043354*	ZmBED21	Nucleus	658	7.6	75232.26	42.51	-0.302
*Zm00001d043856*	ZmBED22	Nucleus	713	8.31	79254.04	46.53	-0.771
*Zm00001d047922*	ZmBED23	Nucleus	183	9.15	21043.04	50.18	-0.611
*Zm00001d049450*	ZmBED24	Nucleus	796	5.83	90980.17	46.67	-0.389
*Zm00001d053219*	ZmBED25	Nucleus	135	5.22	15475.69	56.15	-0.316

The grand average of hydropathicity (GRAVY) values were calculated using the ProtParam tool (Expasy).

### Conserved motifs, protein domains, and gene structures of the ZmBED family

**Figure 1:**
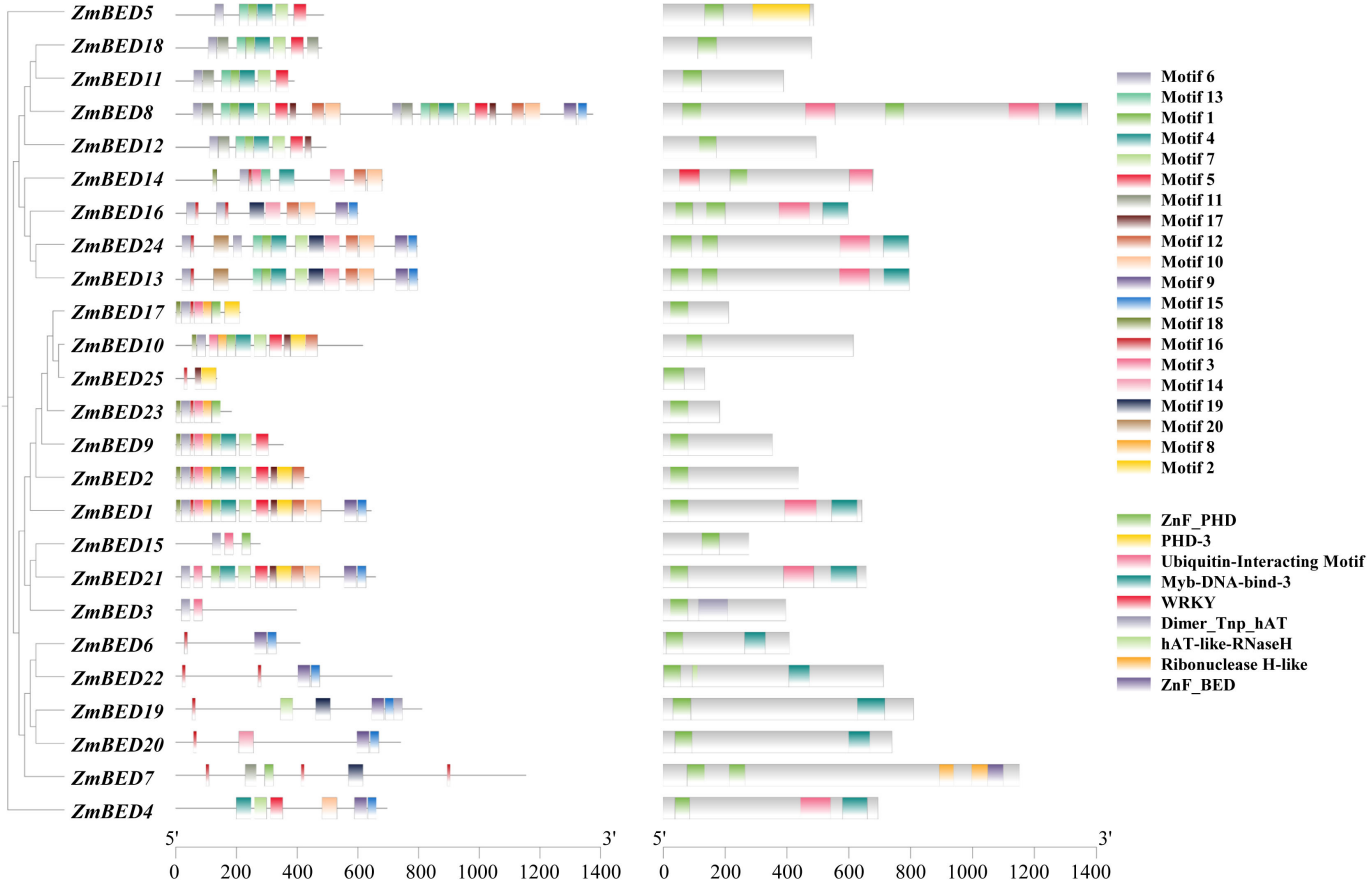
Conserved motifs and domain organization of ZmBED family proteins. Conserved motifs were predicted using MEME (upper panel), and conserved domains were identified using InterPro and NCBI CD-Search (lower panel). Phylogenetic relationships among the 25 ZmBED members are shown on the left. All members contain the Zf-BED domain. Additional domains, including Ribonuclease H-like and Dimer_Tnp_hAT, are present in specific members.

To investigate sequence conservation, conserved motifs in the 25 ZmBED proteins were predicted using the MEME online tool (https://meme-suite.org/meme/tools/meme). The analysis revealed variation in motif abundance among family members. ZmBED3 contained the fewest motifs, whereas the other members exhibited higher motif numbers (**Figure 1**). Phylogenetically related members, including ZmBED5, ZmBED18, and ZmBED11, shared similar motif compositions, indicating consistency between evolutionary relationships and sequence conservation patterns (**Figure 1**).

Conserved domain analysis using InterPro and NCBI CD Search showed that all 25 ZmBED proteins contained the Zf BED domain. Beyond this core domain, specific members harbored additional domains, including a ribonuclease H-like domain and a Dimer_Tnp_hAT domain (**Figure 1**). The ribonuclease H-like domain, known to possess nuclease activity in other contexts, was identified in a subset of ZmBED proteins. The Dimer_Tnp_hAT domain, typically associated with hAT transposase dimerization, was also detected in specific members (**Figure 1**). The presence of these additional domains may hint at potential functional diversification among ZmBED family members; however, this hypothesis remains speculative and requires experimental validation (e.g., nuclease activity assays or transposase functional tests) to be confirmed.

Gene structure analysis was performed using the maize genome annotation file and the “Gene Structure View” function in TBtools (**Supplementary Figure 1**). The number of introns varied substantially across the 25 *ZmBED* genes: *ZmBED23* contained no introns, *ZmBED7* contained nine introns, and the majority of members contained between one and five introns (**Supplementary Figure 1**). This variation in intron number indicates structural heterogeneity within the *ZmBED* gene family.

### Subcellular localization of ZmBED family members

**Figure 2:**
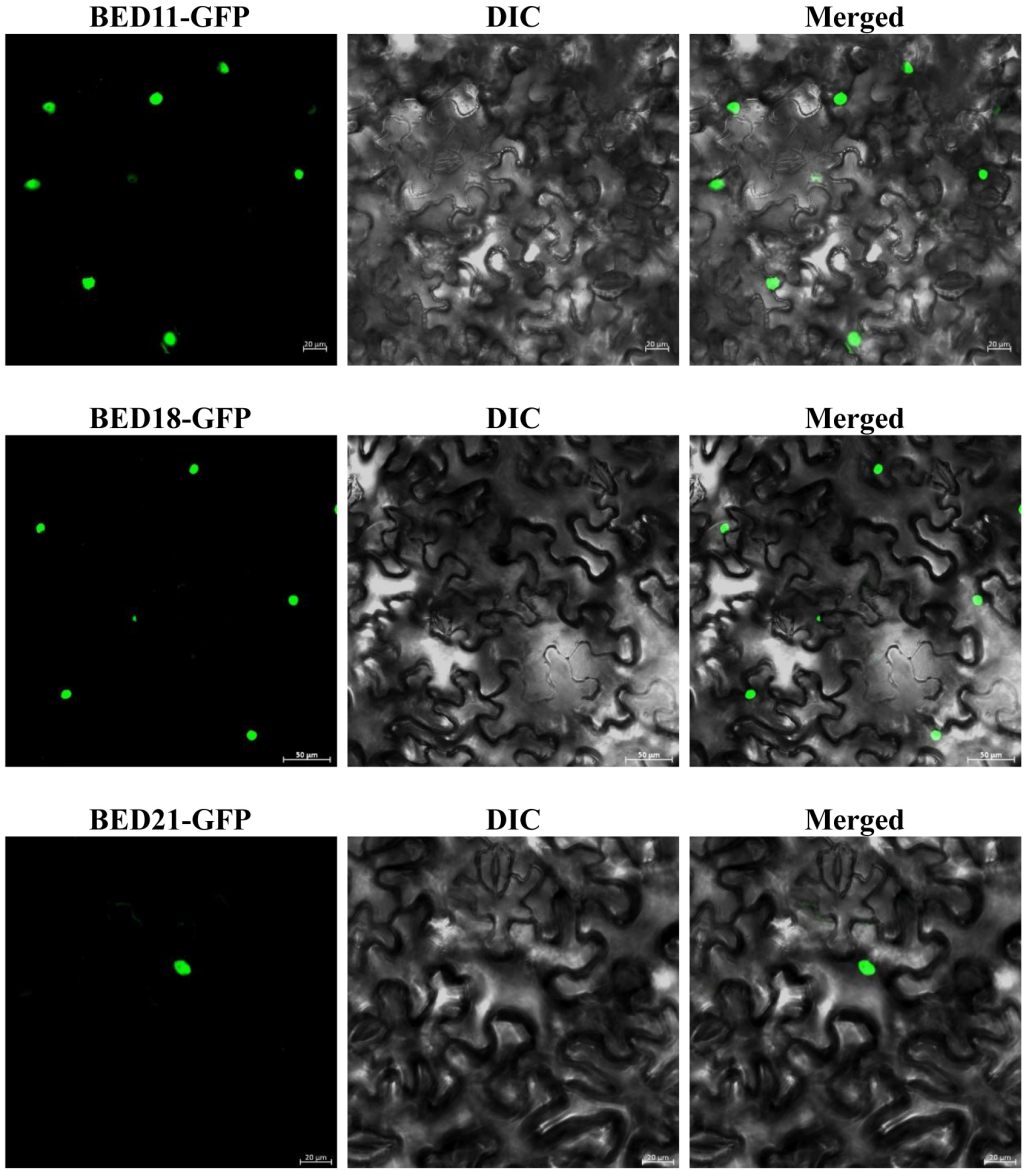
Subcellular localization of ZmBED11, ZmBED18, and ZmBED21 in *Nicotiana benthamiana* epidermal cells. GFP fusion constructs of the ZmBED proteins were transiently expressed using *Agrobacterium*-mediated transformation.

Subcellular localization prediction indicated that ZmBED10 and ZmBED15 are likely targeted to the chloroplast, whereas ZmBED8 exhibits dual localization potential (chloroplast or nucleus) (**Table 1**). All remaining ZmBED family members (ZmBED1~7, 9, 11~14, and 16~25) were predicted to localize to the nucleus (**Table 1**). To experimentally validate these predictions, three members (ZmBED11, 18, and 21) were selected for subcellular localization assays. Fluorescent protein fusion constructs were transiently expressed in *Nicotiana benthamiana* leaf epidermal cells via *Agrobacterium*-mediated transformation. As shown in **Figure 2**, the GFP fusion signals of ZmBED11, ZmBED18, and ZmBED21 were strongly enriched in the nuclear region of tobacco epidermal cells, a pattern distinct from the uniform nucleocytoplasmic distribution of free GFP reported in plant cells (Liu et al., 2022; Sun et al., 2026). Together with the presence of predicted nuclear localization signals (NLSs) in their amino acid sequences, these observations support that the three ZmBED proteins are targeted to the nucleus.

### Phylogenetic analysis across species

**Figure 3:**
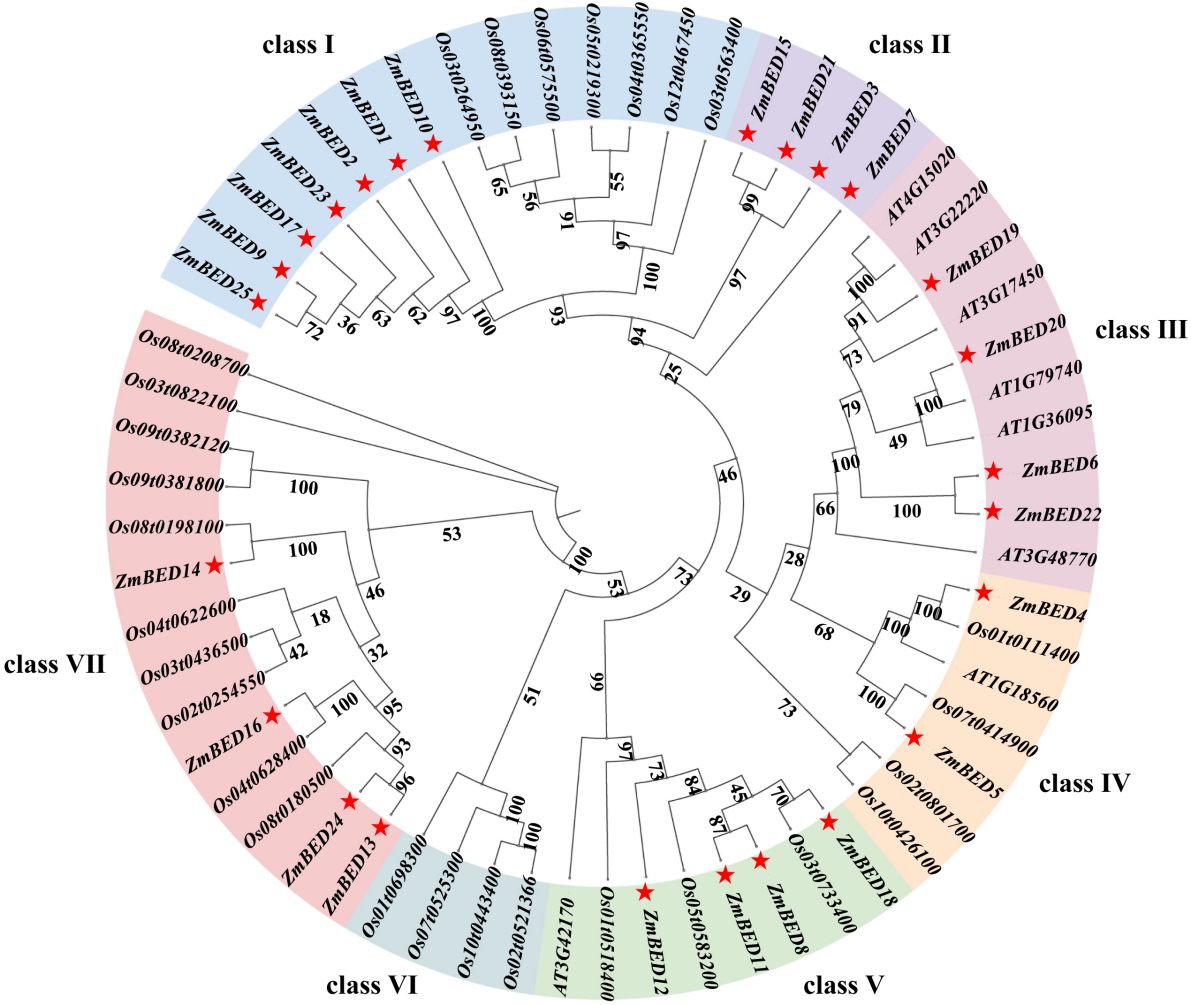
Phylogenetic relationships of Zf-BED domain-containing proteins from maize, rice, and *Arabidopsis thaliana*. A phylogenetic tree was constructed using BED protein sequences from maize (25), rice (28), and *Arabidopsis* (8). Based on tree topology and conserved motif composition, the members were classified into seven groups (I~VII).

To investigate the phylogenetic relationships of Zf-BED domain-containing proteins across different species, a phylogenetic tree was constructed using BED protein sequences from maize (*Zea mays*), *Arabidopsis thaliana*, and rice (*Oryza sativa*). Following domain validation of BLAST-derived sequences, 28 rice proteins and eight Arabidopsis proteins were confirmed to contain the Zf-BED domain. These sequences, together with the 25 ZmBED proteins, were included in the phylogenetic analysis. Based on the phylogenetic tree and conserved motif profiles, all BED family members from the three species were classified into seven distinct clades (Groups I~VII) (**Figure 3**). Among ZmBED members, Group I contained seven proteins; Groups II, III, V, and VII each contained four; and Group IV contained two. Notably, no ZmBED member was assigned to Group VI, which consists exclusively of Arabidopsis and rice BED proteins. This absence may indicate lineage-specific loss or functional divergence of Group VI *BED* genes in maize, although a more comprehensive evolutionary analysis involving additional grass species would be required to confirm this possibility.

### Chromosomal distribution and synteny analysis of ZmBED members

**Figure 4:**
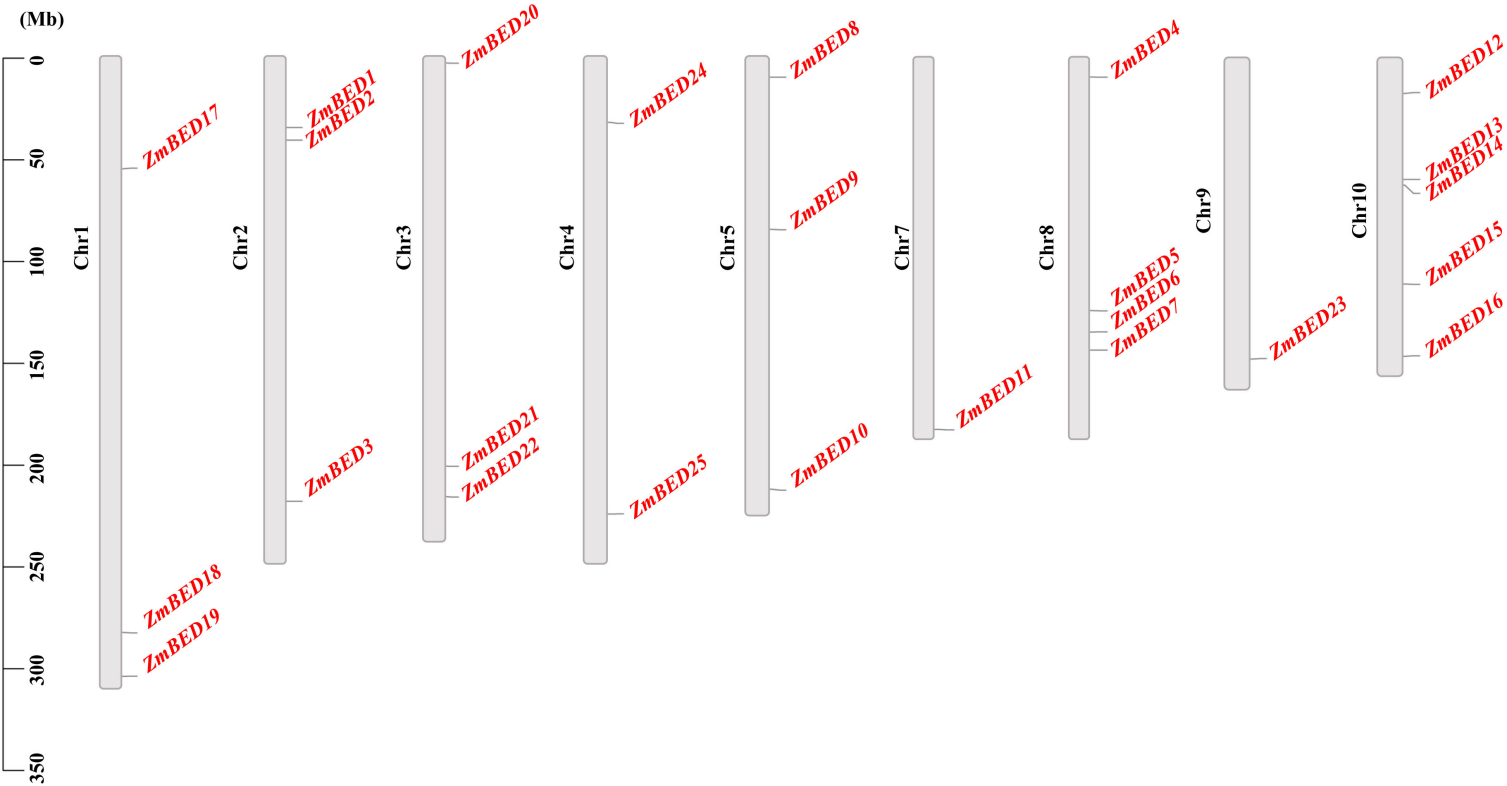
Chromosomal distribution of *ZmBED* members in maize. The genomic positions of 25 *ZmBED* genes were mapped onto maize chromosomes using TBtools. Chromosome numbers are indicated at the top or left of each chromosome. The number of ZmBED members per chromosome is as follows: Chr1 (3), Chr2 (3), Chr3 (3), Chr4 (2), Chr5 (3), Chr6 (0), Chr7 (1), Chr8 (4), Chr9 (1), Chr10 (5). Gene names or identifiers may be placed on the right side of each chromosome, space permitting.

The chromosomal localization of the 25 *ZmBED* family members was visualized using the “Gene Location Visualize from GFF” function in TBtools (**Figure 4**). *ZmBED* genes were distributed on all maize chromosomes except chromosome 6. The number of *ZmBED* members per chromosome was as follows: five on chromosome 10; four on chromosome 8; three on each of chromosomes 1, 2, 3, and 5; two on chromosome 4; and one on each of chromosomes 7 and 9.

To investigate the evolutionary conservation of *BED* family genes across divergent species, interspecies synteny analysis was conducted among maize (*Zea mays*), rice (*Oryza sativa*), and *Arabidopsis thaliana*. The analysis identified syntenic relationships between nine *ZmBED* genes and eight rice *BED* genes (**Supplementary Figure 2**). No syntenic relationships were detected between any *ZmBED* genes and Arabidopsis *BED* genes (**Supplementary Figure 2**). To further investigate the evolutionary relationships of *BED* family genes within maize, intraspecies synteny analysis was performed using the “Advanced Circos” function in TBtools. Syntenic relationships were identified on chromosomes 1, 4, 5, and 10, involving five *ZmBED* genes (**Supplementary Figure 3**).

### *cis*-regulatory element analysis of *ZmBED* promoters

**Figure 5:**
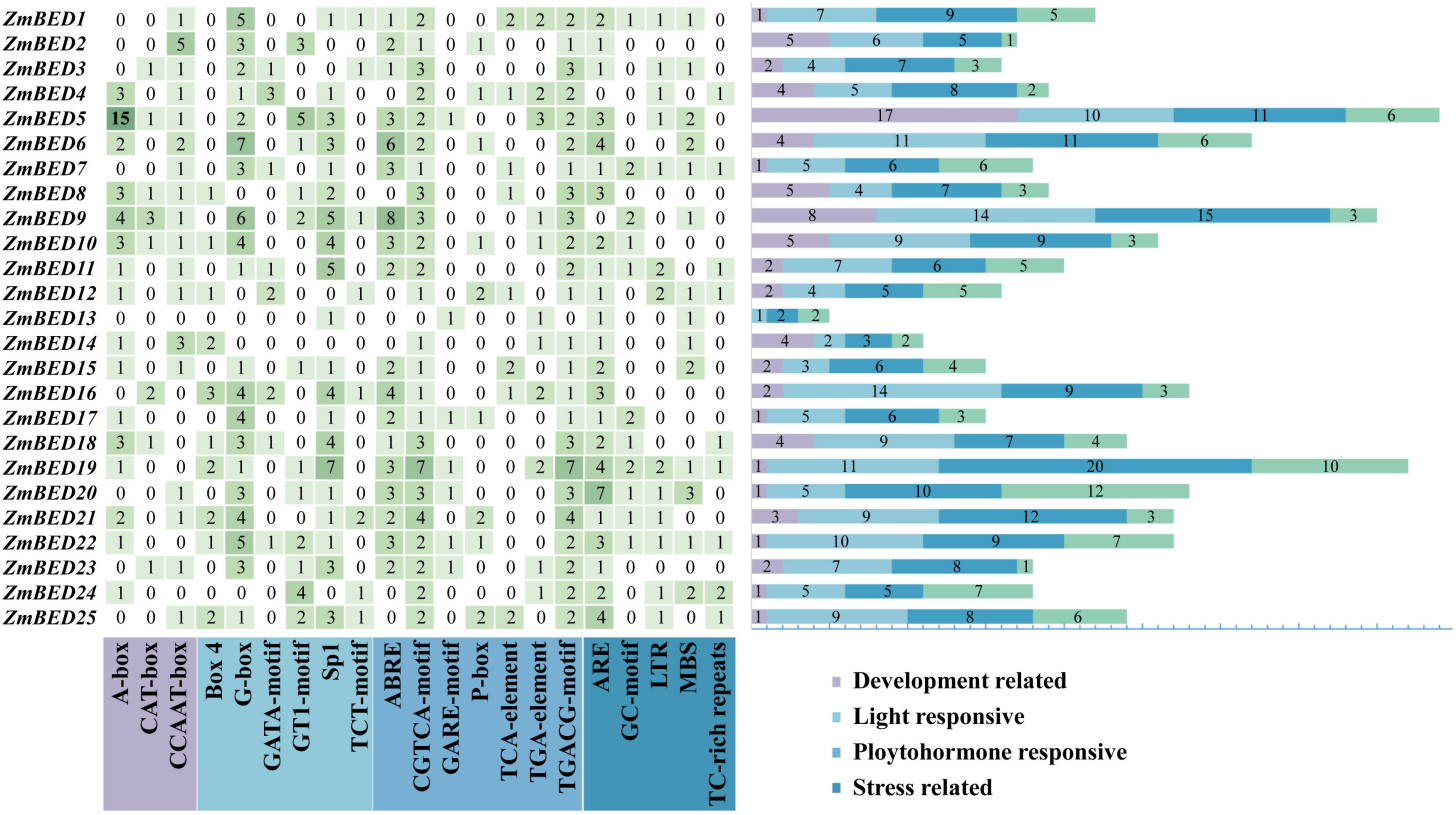
Distribution of *cis*-regulatory elements in *ZmBED* promoters. Promoter sequences (2, 000 bp upstream of the start codon) of the 25 *ZmBED* genes were analyzed using PlantCARE. Bars indicate the number of genes containing each element type: light-responsive (25 genes), anaerobic induction (23 genes), drought-inducible (14 genes), low-temperature response (13 genes), and defense and stress response (9 genes). The total number of elements per type is given in parentheses (e.g., anaerobic induction: 51 elements across 23 genes).

To investigate the potential regulatory mechanisms of *ZmBED* genes, the 2, 000 bp upstream promoter sequences of each family member were extracted using TBtools. *cis*-regulatory elements in these promoter regions were predicted with PlantCARE, and the results were visualized using TBtools. The predicted *cis*-regulatory elements were classified into three categories: (1) hormone-responsive elements (salicylic acid, abscisic acid, auxin, methyl jasmonate, and gibberellin); (2) abiotic stress-responsive elements (light-responsive, drought-inducible, anaerobic induction, low-temperature response, and defense/stress response); and (3) maize growth- and development-related elements (zein metabolism regulation, endosperm expression, meristem expression regulation, and seed-specific regulation) (**Figure 5**).

Quantitative analysis of the predicted *cis*-regulatory elements revealed the following: light-responsive elements were present in the promoters of all 25 *ZmBED* genes (**Figure 5**). Drought-inducible elements were identified in 14 genes (total of 20 elements). Low-temperature response elements were detected in 13 genes (total of 16 elements). Anaerobic induction elements were found in 23 genes (total of 51 elements). Defense and stress response elements were present in nine genes (total of 10 elements). Among all *ZmBED* family members, *ZmBED21* had the highest total number of *cis*-regulatory elements in its promoter region. The majority of *ZmBED* gene promoters contained both abiotic stress-responsive and hormone-responsive elements.

### Protein-protein interaction network prediction of ZmBED members

To investigate potential interacting partners of ZmBED proteins, protein-protein interactions (PPIs) were predicted using the STRING database. The predicted PPI network comprised 90 interacting proteins and 16 ZmBED members (**Supplementary Figure 4**). Within the predicted network, ZmBED4 exhibited the highest node degree, with 60 potential direct interactions. Among the non-ZmBED proteins in the network, protein A0A096QBS7 showed the highest node degree. Domain analysis revealed that protein A0A096QBS7 contains a SWIM-type zinc finger domain. Additionally, the CCR1 protein, which also displayed a high node degree, contains a protein kinase domain characteristic of the serine/threonine protein kinase family.

### Expression profiling of *ZmBED* members

**Figure 6:**
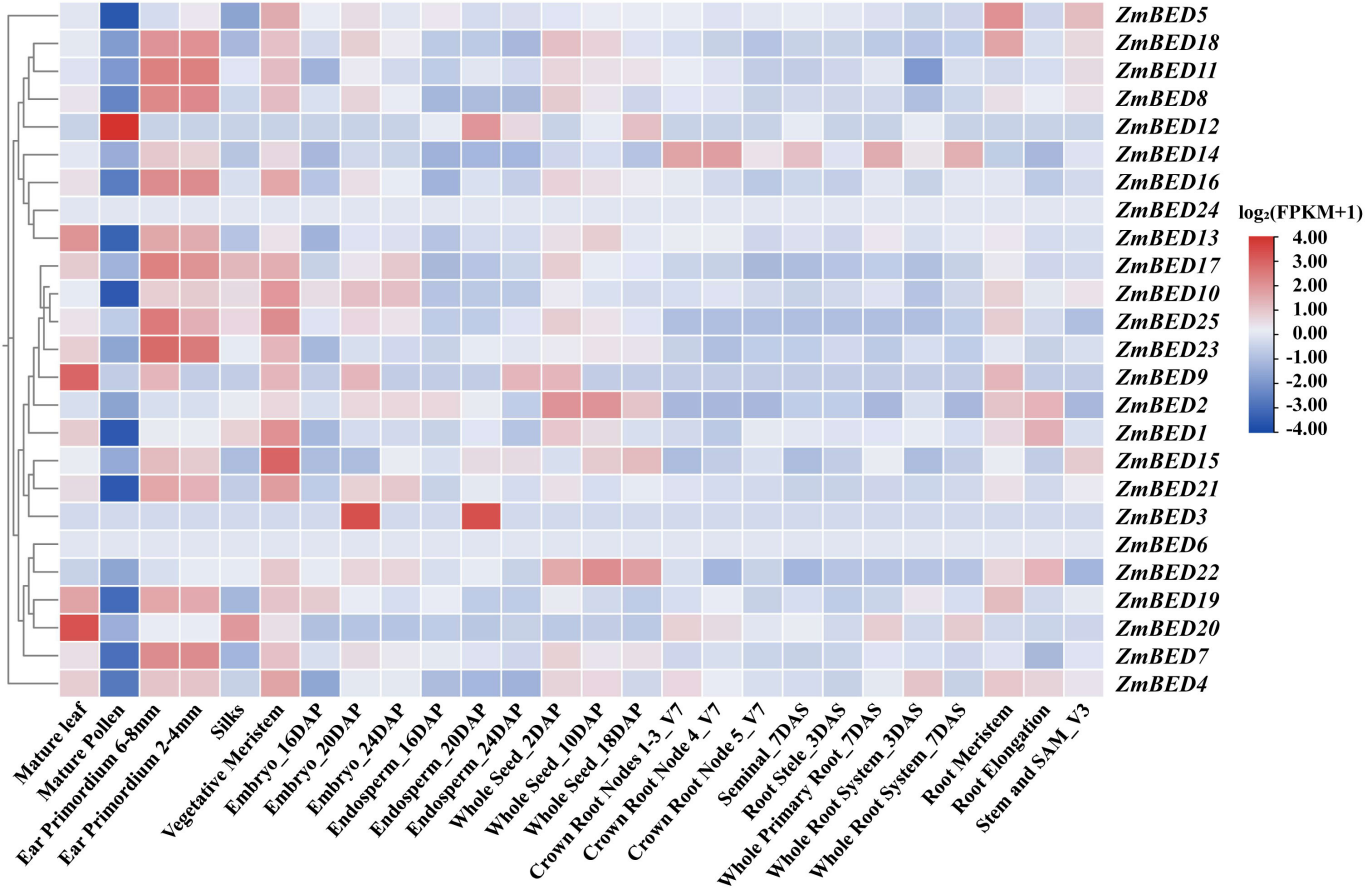
Spatiotemporal expression patterns of *ZmBED* members. Expression data were obtained from the qTeller transcriptome database (maizeGDB). The heatmap displays expression levels across various tissues and developmental stages (as indicated in the figure). The color scale represents normalized expression values [log_2_(FPKM + 1)], with red indicating high expression and blue indicating low expression.

To investigate the expression patterns of *ZmBED* family members, a spatiotemporal expression heatmap was generated based on qTeller transcriptome data from the MaizeGDB database (**Figure 6**). *ZmBED12* exhibited high expression in mature pollen but low expression in all other examined tissues. *ZmBED3* showed high expression in embryo and endosperm tissues at 20 days after pollination and low expression in other tissues. *ZmBED9*, *13*, and *20* displayed high expression levels in mature leaves. In inflorescence primordia and vegetative meristems, more *ZmBED* genes showed elevated expression levels compared with other tissues.

### Expression of *ZmBED* genes under abiotic stress conditions

**Figure 7:**
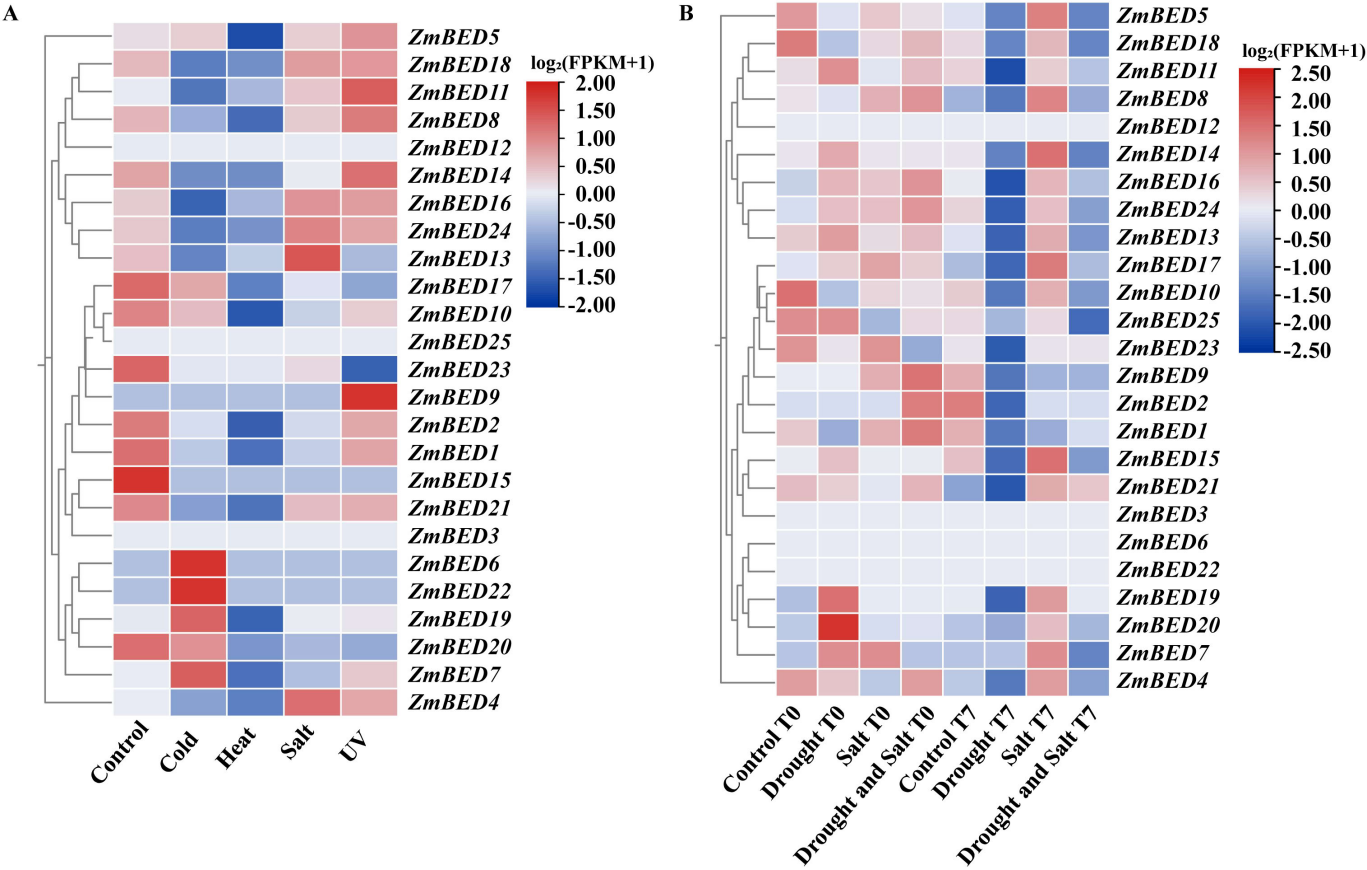
Expression of *ZmBED* genes under abiotic stresses and recovery. **(A)** Heatmap showing expression changes of *ZmBED* genes under cold, heat, salt, and UV stress. Upregulated genes are indicated in red, and downregulated genes in blue. **(B)** Expression dynamics under drought, salt, combined stress (T0), and after a 7-day recovery period (T7). The color scale indicates log_2_-transformed fold change in transcript abundance (relative to control), with red indicating high expression and blue indicating low expression.

To investigate the transcriptional responses of maize *ZmBED* genes to abiotic stresses, their expression profiles were examined under cold, heat, salt, and UV stress conditions (**Figure 7A**). Under cold stress, six *ZmBED* genes (*ZmBED6*, *22*, *7*, *19*, *17*, and *20*) exhibited elevated transcript levels. Among these, *ZmBED6* and *22* showed the highest expression levels across the entire gene family (**Figure 7A**). In contrast, heat stress led to a general downregulation of most *ZmBED* genes. Under salt stress, nine *ZmBED* genes (*ZmBED5*, *11*, *21*, *8*, *4*, *16*, *18*, *24*, and *13*) were upregulated, with *ZmBED13* displaying the highest relative expression level. Under UV stress, 14 *ZmBED* genes were induced, including *ZmBED5*, *10*, *1*, *2*, *9*, *11*, *21*, *8*, *14*, *4*, *16*, *18*, *24*, and *7*; among these, *ZmBED9* exhibited the most pronounced upregulation (**Figure 7A**).

To further assess expression dynamics under water-related stresses and post-stress recovery, transcript levels of *ZmBED* genes were analyzed under drought stress, salt stress, combined drought-salt stress, and after a seven-day recovery period (T7) following each 10-day stress treatment (T0) (**Figure 7B**). Under drought stress, *ZmBED20* showed a significant increase in expression at T0 (e.g., fold change > 2.0, *P* < 0.05), which returned to near-baseline levels after the seven-day recovery period (T7). By contrast, under salt stress, several salt-induced *ZmBED* genes did not exhibit a substantial decrease in expression following recovery, a pattern distinct from that observed under drought or combined drought-salt conditions (**Figure 7B**).

### Phenotypic, transcriptional, and rhizosheath responses of maize to moderate drought stress

To evaluate the effects of moderate drought stress on maize growth, a controlled soil water content experiment was conducted. At day 0 (pre-treatment), no phenotypic differences were observed between the control and drought-treated groups; plants in both groups had expanded, dark-green leaves and showed uniform growth (**Supplementary Figures 5A, B**). After 14 days of treatment, plants in the well-watered control group maintained normal growth, with erect leaves and typical green coloration (**Supplementary Figure 5C**). In contrast, plants subjected to drought stress (50% relative soil water content) displayed distinct stress-induced phenotypes, including leaf curling, drooping, chlorotic leaf tips, and overall growth inhibition (**Supplementary Figure 5D**). These phenotypic differences confirm that the moderate drought treatment effectively imposed growth-limiting conditions, providing a valid basis for subsequent gene expression and rhizosheath analyses.

### Expression profiles of *ZmBED* genes under drought stress

**Figure 8:**
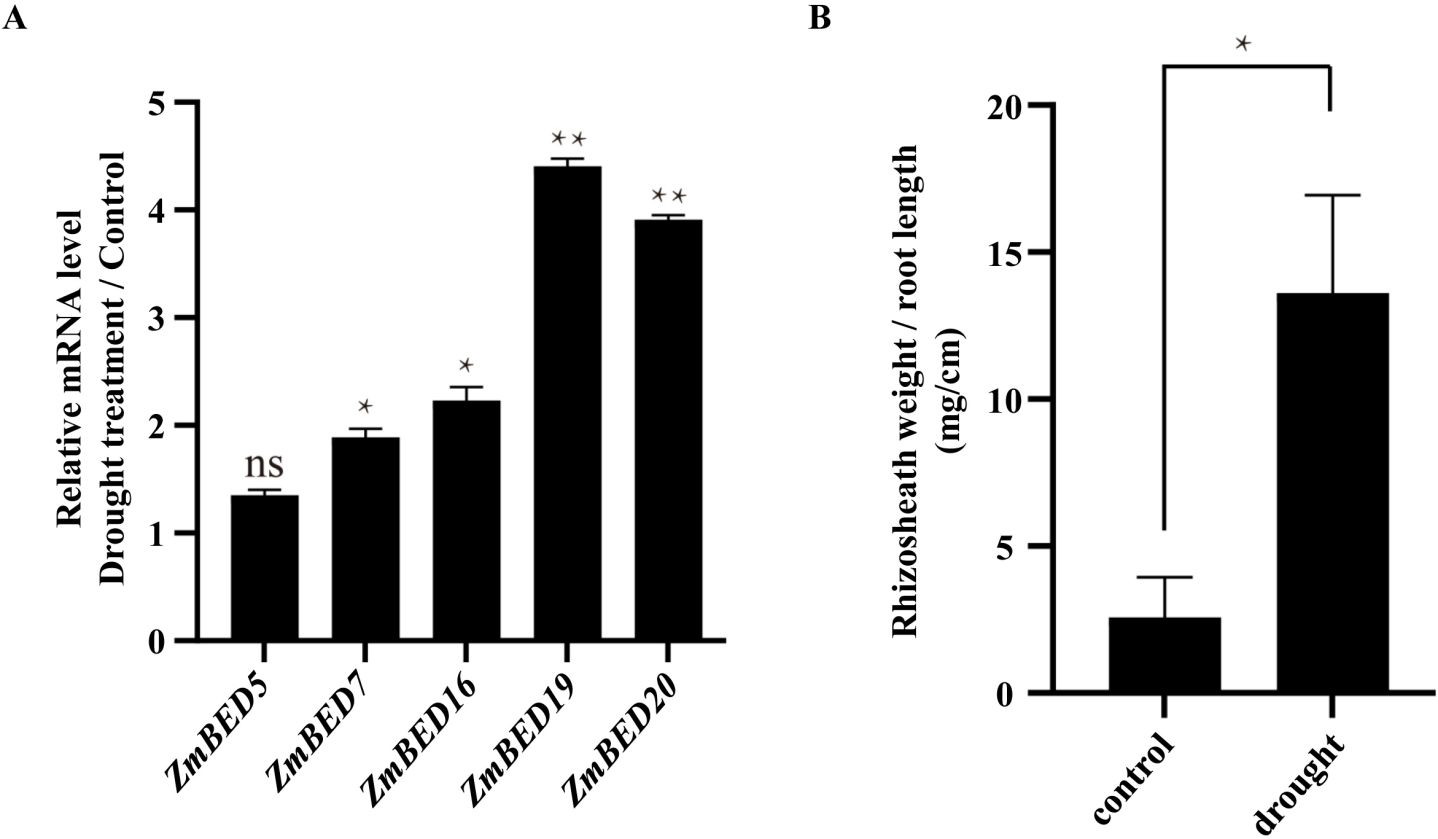
Drought-induced expression of *ZmBED* genes and changes in rhizosheath weight per root length in maize B73 inbred line. **(A)** Expression levels of *ZmBED5*, *7*, *16*, *19*, and *20* under drought treatment detected by RT-qPCR. Expression values were normalized to *ZmActin* (*GRMZM2G126010*) as the internal reference gene and calculated using the 2^^–ΔΔCt^ method. **(B)** Evaluation of rhizosheath weight (mg) and root length (cm) in the B73 inbred line under well−watered and drought treatment conditions. Statistical analysis of the ratio of rhizosheath weight to root length (unit: mg/cm) under control and drought stress treatments in maize. Bars represent mean ± SD of three biological replicates. Asterisks indicate statistically significant differences compared with the control group (two-tailed unpaired Student’s *t*-test, *P* < 0.05).

The transcript levels of five *ZmBED* genes (*ZmBED5*, *7*, *16*, *19*, and *20*) were quantified in root and rhizosheath tissues under control and drought conditions using RT-qPCR based on their significant differential expression in the RNA-seq analysis (**Figure 7B**, 8A). Under drought stress, *ZmBED7*, *16*, *19*, and *20* were significantly upregulated compared with control conditions (**Figure 8A**). This upregulation pattern was consistent with the transcriptome data presented online in **Figure 7B**. In contrast, *ZmBED5* did not exhibit a statistically significant change in expression under drought stress. These results demonstrate that four of the five *ZmBED* genes examined are transcriptionally responsive to drought in maize root-associated tissues.

To determine whether drought stress alters rhizosheath development, rhizosheath weight per unit root length was measured after 14 days of treatment (**Figure 8B**). Under control conditions, the mean rhizosheath weight was 12.3 mg·cm^-1^ (rhizosheath mass per unit root length). Following drought exposure, the mean value increased to 21.7 mg·cm^-1^, representing a 76.4% increase, and the difference between groups was statistically significant. These results indicate that moderate drought stress promotes rhizosheath accumulation in maize. Under drought stress, the upregulation of *ZmBED7*, *16*, *19*, and *20* coincided with increased rhizosheath mass. Whether these genes play any direct role in rhizosheath development requires further experimental validation.

## Discussion

In this study, we systematically identified 25 *ZmBED* genes in the maize genome. Through phylogenetic, structural, and expression analyses, we characterized their evolutionary conservation and divergence and explored their potential involvement in abiotic stress responses, particularly drought. Our results provide a foundational framework for functional dissection of the *ZmBED* family in maize.

### Evolutionary conservation and lineage-specific expansion of the *ZmBED* family

The Zf-BED domain is a conserved DNA-binding module first characterized in the Boundary Element Associated Factor and transposases of *Drosophila* (Smit, 1999). Subsequent studies have extended its functional repertoire to include protein–protein interactions, subcellular localization regulation, and integration into immune signaling pathways (Zhou et al., 2017; Ma et al., 2024). In plants, the SLEEPER gene family in *Arabidopsis thaliana* represents an angiosperm-specific group derived from *hAT* transposases via retrotransposition, providing direct evidence for the domestication of transposable element-derived sequences into host functional genes (Knip et al., 2012). Consistent with this evolutionary model, our study revealed substantial diversity among ZmBED members in protein length (135–1, 375 aa) and molecular weight (15.4–154.3 kDa), with all members retaining the Zf-BED domain. Notably, 21 of the 25 ZmBED proteins contained conserved motif 1, suggesting a degree of functional conservation across the family.

Phylogenetic analysis classified BED proteins from maize, rice, and *Arabidopsis* into seven distinct subfamilies. We identified nine orthologous gene pairs between maize and rice (both Poaceae), but no syntenic relationships with *Arabidopsis*. These findings indicate significant evolutionary divergence of *BED* genes between monocots and dicots, supporting the notion of lineage-specific expansions and functional specialization following the separation of these two angiosperm lineages.

### Domain architecture suggests origins from intact *hAT* transposons

A subset of ZmBED members harbored additional domains, including RNase H-like and Dimer_Tnp_hAT domains, alongside the canonical Zf-BED domain. The RNase H-like domain constitutes the catalytic core of many transposases, mediating DNA strand cleavage and transfer (Lannes et al., 2023), whereas the Dimer_Tnp_hAT domain facilitates transposase dimerization or multimerization, a critical step in transposition complex assembly (Knip et al., 2012). The retention of these domains in ZmBED proteins strongly suggests that they originated from intact *hAT* transposons. Over evolutionary time, these proteins likely underwent domain recombination and functional divergence, acquiring novel roles in plant development and stress adaptation.

Interestingly, in the snapdragon *Tam3* transposase system, the BED domain exhibits a dual function in plasma membrane anchoring and DNA recognition. Under high-temperature conditions, the BED domain anchors the protein to the plasma membrane via N-terminal aromatic amino acids, preventing nuclear import; under low-temperature conditions, this anchoring is relieved, allowing nuclear entry and transposition (Zhou et al., 2017). Whether a similar temperature-responsive regulatory mechanism operates in ZmBED proteins remains an open question for future investigation.

### Putative roles in stress signaling via phytohormone pathways

In plant immunity, BED domains function as integrated domains (IDs) within BED-NLR proteins, where a BED domain is fused into the N-terminal coiled-coil (CC) domain, forming a CC-BED module (Ma et al., 2024). Several BED-NLR resistance genes have been functionally characterized, including the wheat stripe rust resistance genes *Yr5*/*Yr7*/*YrSP* (Marchal et al., 2018), the wheat powdery mildew resistance gene *Pm6Sl* (Ma et al., 2024), and the barley leaf rust resistance gene *Rph15* (Chen et al., 2021a). In Pm6Sl, the CC-BED module is both necessary and sufficient for conferring resistance to powdery mildew. Our promoter analysis revealed that *ZmBED* genes contain abundant *cis*-regulatory elements responsive to abscisic acid (ABA), methyl jasmonate (MeJA), and salicylic acid (SA), suggesting their potential involvement in both biotic and abiotic stress responses via phytohormone signaling pathways. These observations place the *ZmBED* family within a broader regulatory landscape where transposon-derived domains have been co-opted into plant stress signaling networks.

### Limitations and future perspectives

Despite these insights, several limitations of this study should be acknowledged. First, our conclusions are primarily derived from bioinformatic predictions and transcriptomic data; functional validation through overexpression or CRISPR/Cas9-mediated knockout is necessary to definitively assign biological roles to individual *ZmBED* genes. Second, the protein-protein interaction networks involving ZmBED members, as well as their potential crosstalk with established stress-related transcription factor families (e.g., NAC, AP2/ERF, bHLH), remain unexplored. Third, the downstream target genes regulated by ZmBED proteins have yet to be identified, for instance through ChIP-seq or DAP-seq. Fourth, this study did not address the potential functions of *ZmBED* genes in biotic stress responses or normal developmental processes, despite accumulating evidence that plant Zf-BED proteins participate in pathogen resistance (Marchal et al., 2018; Chen et al., 2021a; Ma et al., 2024).

A particularly promising avenue for future research concerns the maize rhizosheath. To date, however, no studies have functionally linked BED-family genes to rhizosheath formation in any plant species. Our RT-qPCR and rhizosheath weight measurements revealed that four ZmBED genes (*ZmBED7*, *16*, *19*, and *20*) were coordinately upregulated under drought stress, concomitant with a marked increase in rhizosheath mass (**Figure 8**). Notably, transcriptomic data indicated that *ZmBED7* and *ZmBED16* are preferentially expressed in early primary roots (**Supplementary Figure 6**), consistent with a potential role in root hair initiation. Based on their expression patterns and the known roles of BED-domain proteins in transcriptional regulation, we propose a tentative model in which the coordinated upregulation of these genes may modulate root hair elongation-related genes (e.g., *RSL*-class transcription factors), thereby indirectly contributing to root epidermal differentiation and providing a physical scaffold for rhizosheath formation. However, we emphasize that this model remains hypothetical and is primarily grounded in correlative evidence. Future work should focus on root hair morphological characterization, spatiotemporal expression profiling in root/rhizosheath tissues, and genetic modulation of these candidates to establish their causal roles and underlying mechanisms.

## Conclusion

In summary, this study presents a comprehensive genome-wide identification and systematic characterization of the maize *ZmBED* gene family. We have elucidated the evolutionary characteristics, domain architectures, and abiotic stress response patterns of this family, and have identified a set of candidate genes responsive to drought and other abiotic stresses. These findings expand our understanding of how transposon-derived BED domains have been repurposed for plant stress adaptation and provide valuable genetic resources for the future improvement of abiotic stress tolerance in maize, and potentially in other cereal crops.

## Data Availability

The original contributions presented in the study are included in the article/**Supplementary Material**. Further inquiries can be directed to the corresponding authors.

## References

[B1] Ali M. A. Younis S. Wallerman O. Gupta R. Andersson L. Sjöblom T. (2015). Transcriptional modulator ZBED6 affects cell cycle and growth of human colorectal cancer cells. Proc. Natl. Acad. Sci. U.S.A. 112, 7743–7748. doi: 10.1016/j.canlet.2016.04.020 26056301 PMC4485122

[B2] Babu M. M. Iyer L. M. Balaji S. Aravind L. (2006). The natural history of the WRKY-GCM1 zinc fingers and the relationship between transcription factors and transposons. Nucleic Acids Res. 34, 6505–6520. doi: 10.1093/nar/gkl888 17130173 PMC1702500

[B3] Berg J. M. Shi Y. G. (1996). The galvanization of biology: A growing appreciation for the roles of zinc. Science 271, 1081–1085. doi: 10.1126/science.271.5252.1081 8599083

[B4] Cao S. K. Liu R. Wang M. Sun F. Sayyed A. Shi H. . (2022). The small PPR protein SPR2 interacts with PPR-SMR1 to facilitate the splicing of introns in maize mitochondria. Plant Physiol. 190, 1763–1776. doi: 10.1093/plphys/kiac379 35976145 PMC9614438

[B5] Chen C. Chen H. Zhang Y. Thomas H. R. Frank M. H. He Y. . (2020). TBtools: an integrative toolkit developed for interactive analyses of big biological data. Mol. Plant 13, 1194–1202. doi: 10.1016/j.molp.2020.06.009 32585190

[B6] Chen C. Jost M. Clark B. Martin M. Matny O. Steffenson B. J. . (2021a). BED domain‐containing NLR from wild barley confers resistance to leaf rust. Plant Biotechnol. J. 19, 1206–1215. doi: 10.1111/pbi.13542 33415836 PMC8196641

[B7] Chen M. X. Mei L. C. Wang F. Boyagane Dewayalage I. K. W. Yang J. F. Dai L. . (2021b). PlantSPEAD: a web resource towards comparatively analysing stress-responsive expression of splicing-related proteins in plant. Plant Biotechnol. J. 19, 227–229. doi: 10.1111/pbi.13486 33010087 PMC7868970

[B8] Chen C. Wu Y. Li J. Wang X. Zeng Z. Xu J. . (2023). TBtools-II: A "one for all, all for one" bioinformatics platform for biological big-data mining. Mol. Plant 16, 1733–1742. doi: 10.1016/j.molp.2023.09.010 37740491

[B9] Duell R. W. Peacock G. R. (1985). Rhizosheaths on mesophytic grasses. Crop Sci. 25, 880–883. doi: 10.2135/cropsci1985.0011183X002500050036x

[B10] Gu X. Y. Li P. H. Gao X. H. Ru Y. Xue C. Zhang S. J. . (2024). RNA 5-methylcytosine writer NSUN5 promotes hepatocellular carcinoma cell proliferation via a ZBED3-dependent mechanism. Oncogene 43, 624–635. doi: 10.1038/s41388-023-02931-z 38182896 PMC10890930

[B11] Knip M. Pater S. D. Hooykaas P. J. (2012). The SLEEPER genes: a transposase-derived angiosperm-specific gene family. BMC Plant Biol. 12, 192. doi: 10.1186/1471-2229-12-192 23067104 PMC3499209

[B12] Kubo K. Sakamoto A. Kobayashi A. Rybka Z. Kanno Y. Nakagawa H. . (1998). Cys2/His2 zinc-finger protein family of petunia: evolution and general mechanism of target-sequence recognition. Nucleic Acids Res. 26, 608–615. doi: 10.1093/nar/26.2.608 9421523 PMC147284

[B13] Lannes L. Furman C. M. Hickman A. B. Dyda F. (2023). Zinc-finger BED domains drive the formation of the active Hermes transpososome by asymmetric DNA binding. Nat. Commun. 14, 4470. doi: 10.1038/s41467-023-40210-3 37491363 PMC10368747

[B14] Li B. Z. Liu R. N. Zhang H. Liu J. Tian Y. N. Wang P. T. . (2025a). The transcription factor ZmMYB56 regulates high CO_2_‐induced stomatal closure in maize seedlings by regulating ZmHLT1. Plant Cell Environ. 48, 338–349. doi: 10.1111/pce.15137 39262203

[B15] Li Z. Liu C. Zhang Y. Wang B. Ran Q. Zhang J. . (2019). The bHLH family member ZmPTF1 regulates drought tolerance in maize by promoting root development and abscisic acid synthesis. J. Exp. Bot. 70, 5471–5486. doi: 10.1093/jxb/erz307 31267122 PMC6793450

[B16] Li Q. Liu W. Zou H. (2026). ZmWRKY122 enhances waterlogging tolerance in maize via direct activation of ZmPRX101. Crop J. 14 (3), 799–808. doi: 10.1016/j.cj.2025.12.020 38826717

[B17] Li D. Zhang C. X. Dong Z. C. Yang L. H. Wang H. Wang X. . (2025b). ZmDREB1A regulates myo-Inositol-1-phosphate synthase 2 controlling maize germination at low temperatures. J. Agric. Food. Chem. 73, 7562–7573. doi: 10.1021/acs.jafc.4c10477 40111445

[B18] Liu S. T. Shi H. B. Yan L. N. Jiang C. Zhao H. C. Lu H. B. . (2025). Identification of candidate genes and proteins for tasseling stage drought tolerance through integrated transcriptomic and proteomic analysis approach in maize. BMC Plant Biol. 25, 1344. doi: 10.1186/s12870-025-07264-5 41062975 PMC12506335

[B19] Liu H. Xiu Z. Yang H. Ma Z. Yang D. Wang H. . (2022). Maize Shrek1 encodes a WD40 protein that regulates pre-rRNA processing in ribosome biogenesis. Plant Cell 34, 4028–4044. doi: 10.1016/b978-0-323-96102-8.00002-4 35867001 PMC9516035

[B20] Lu Y. Wang K. Ngea G. L. N. Godana E. A. Ackah M. Dhanasekaran S. . (2024). Recent advances in the multifaceted functions of Cys2/His2-type zinc finger proteins in plant growth, development, and stress responses. J. Exp. Bot. 75, 5501–5520. doi: 10.1093/jxb/erae278 38912636

[B21] Ma H. Z. Liu C. Li Z. X. Ran Q. J. Xie G. N. Wang B. M. . (2018). ZmbZIP4 contributes to stress resistance in maize by regulating ABA synthesis and root development. Plant Physiol. 178, 753–770. doi: 10.1104/pp.18.00436 30126870 PMC6181033

[B22] Ma C. Tian X. Dong Z. Li H. Chen X. Liu W. . (2024). An Aegilops longissima NLR protein with integrated CC-BED module mediates resistance to wheat powdery mildew. Nat. Commun. 15, 8281. doi: 10.1038/s41467-024-52670-2 39333612 PMC11436982

[B23] Marchal C. Zhang J. Zhang P. Fenwick P. Steuernagel B. Adamski N. M. . (2018). BED-domain-containing immune receptors confer diverse resistance spectra to yellow rust. Nat. Plants 4, 662–668. doi: 10.1038/s41477-018-0236-4 30150615

[B24] Moreau E. L. P. Maughan P. J. Spanner R. Jellen E. N. Moscou M. J. Kianian S. (2026). Mapping Pc94-mediated crown rust resistance in oat reveals a zfBED NLR and introgression variation. bioRxiv. doi: 10.64898/2026.01.23.701375

[B25] Park S. R. Jeong Y. Son S. (2025). Functions of transcription factor superfamilies in rice immunity. Crop J. 13, 5–22. doi: 10.1016/j.cj.2024.10.006 38826717

[B26] Salika R. Riffat J. (2021). Abiotic stress responses in maize: a review. Acta Physiol. Plant 43, 130. doi: 10.1007/s11738-021-03296-0 30311153

[B27] Schwechheimer C. Bevan M. (1998). The regulation of transcription factor activity in plants. Trends Plant Sci. 3, 378–383. doi: 10.1016/s1360-1385(98)01302-8

[B28] Shi W. Sun Q. Zhao Y. Wang Y. Liu Y. Li C. . (2026). ZmSNAC1 cooperates with its interaction partner ZmNAC19 and ZmCAR11 to regulate drought tolerance in maize. Plant Physiol. Biochem. 231, 110985. doi: 10.1016/j.plaphy.2025.110985 41455433

[B29] Smit A. F. A. (1999). Interspersed repeats and other mementos of transposable elements in mammalian genomes. Curr. Opin. Genet. Dev. 9, 657–663. doi: 10.1016/s0959-437x(99)00031-3 10607616

[B30] Song Y. C. Chen M. X. Zhang K. L. Reddy A. S. N. Cao F. L. Zhu F. Y. (2023). QuantAS: a comprehensive pipeline to study alternative splicing by absolute quantification of splice isoforms. New Phytol. 240, 928–939. doi: 10.1111/nph.19193 37596706

[B31] Steiner F. A. Wild A. J. Tyborski N. Tung S. Y. Koehler T. Buegger F. . (2024). Rhizosheath drought responsiveness is variety‐specific and a key component of belowground plant adaptation. New Phytol. 242, 479–492. doi: 10.1111/nph.19638 38418430

[B32] Sun F. Jiang P. P. Liu S. Zhang X. Wu N. Liu W. . (2026). Maize ZmBCCIP facilitates cleavage at the A3 site in pre‐rRNA processing and is crucial to seed development and vegetative growth. Plant J. 125, e70719. doi: 10.1111/tpj.70719 41616378

[B33] Wang F. Chen Y. Yang R. S. Luo P. Wang H. W. Zhang R. Z. . (2025). Identification of ZmSNAC06, a maize NAC family transcription factor with multiple transcripts conferring drought tolerance in Arabidopsis. Plants 14, 12. doi: 10.1109/icmtma.2015.136 39795271 PMC11722792

[B34] Yoshihisa A. Yoshimura S. Zhou J. Nishikawa K. Yamaguchi K. Kawasaki T. (2025). Essential role of rice ERF101 in the perception of TAL effectors and immune activation mediated by the CC-BED NLR Xa1. Plant Cell Rep. 44, 49. doi: 10.1007/s00299-025-03436-7 39907826 PMC11799121

[B35] Zhou H. Hirata M. Osawa R. Fujino K. Kishima Y. (2017). Detainment of Tam3 transposase at plasma membrane by its BED-zinc finger domain. Plant Physiol. 173, 1492–1501. doi: 10.1104/pp.16.00996 28008001 PMC5291012

[B36] Zuluaga A. P. Bidzinski P. Chanclud E. Ducasse A. Cayrol B. Selvaraj M. G. . (2020). The rice DNA-binding protein ZBED controls stress regulators and maintains disease resistance after a mild drought. Front. Plant Sci. 11, 1265. doi: 10.3389/fpls.2020.01265 33013945 PMC7461821

