# Supplementary material for: Genome-wide identification and characterization of the ZmBED gene family in maize and its putative roles in drought adaptation via rhizosheath formation
Source: Front Plant Sci. 2026 Jul 7;17:1884999. doi: 10.3389/fpls.2026.1884999 (PMC13385653; doi:10.3389/fpls.2026.1884999)
Supplement: Supplementary file 1 [file DataSheet1.pdf]

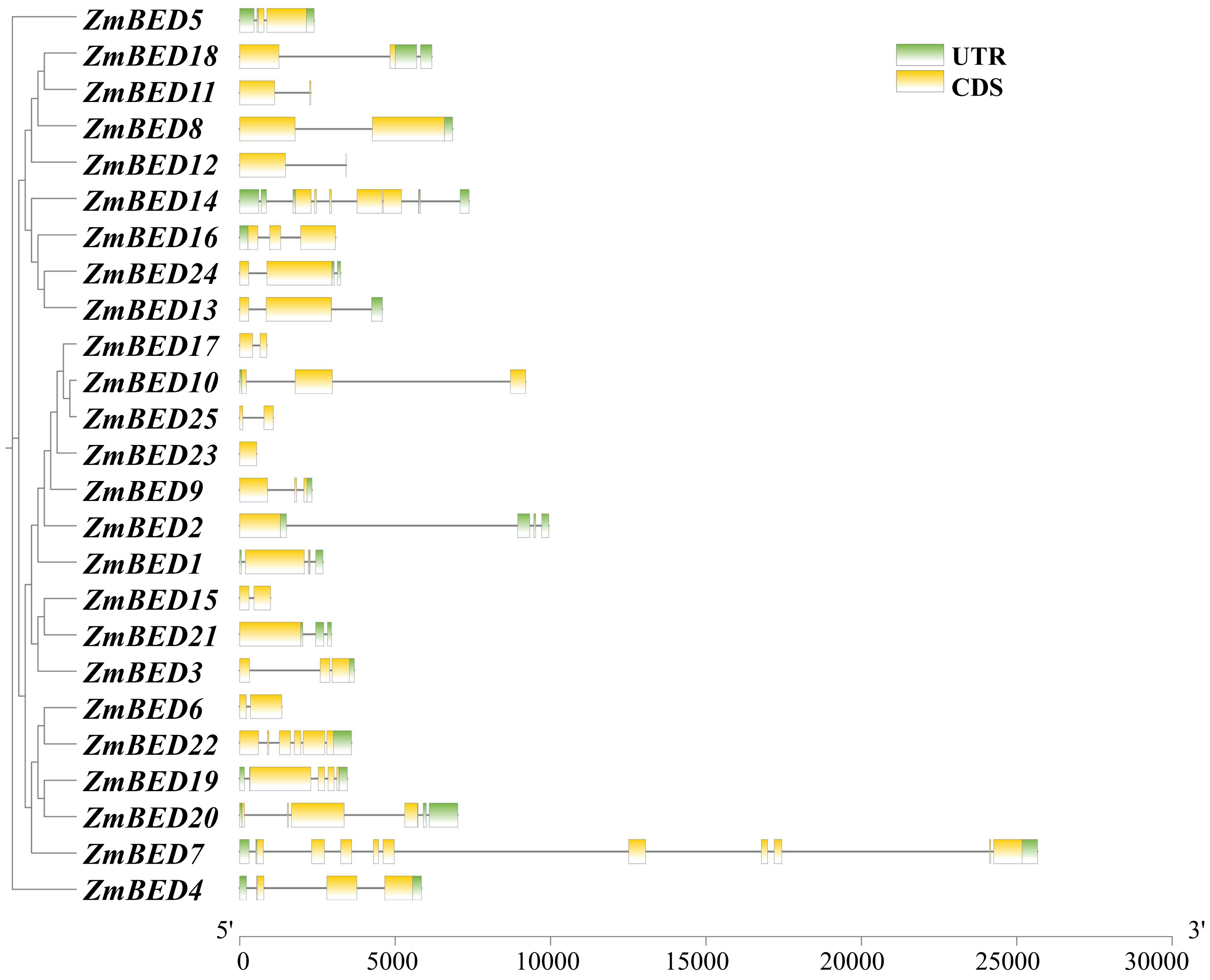

Supplementary Figure 1 Gene structures of *ZmBED* family members.

Exon intron organization was visualized using TBtools based on the maize genome annotation. Yellow boxes represent exons, and black lines represent introns. The number of introns ranges from 0 (*ZmBED23*) to 9 (*ZmBED7*), with most members containing 1~5 introns.

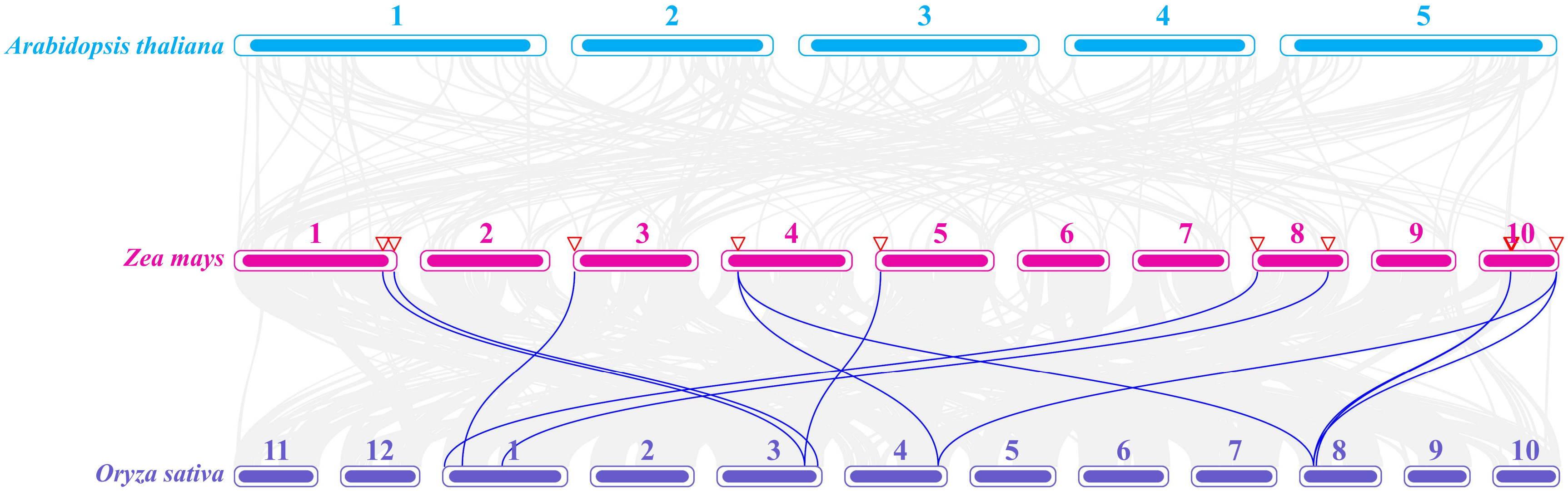

Supplementary Figure 2 Interspecies synteny analysis of *BED* genes among maize, rice, and *Arabidopsis thaliana*.
Syntenic relationships are shown between nine *ZmBED* genes and eight rice *BED* genes. No syntenic relationships were observed between maize and *Arabidopsis BED* genes. Each syntenic pair is connected by a line.

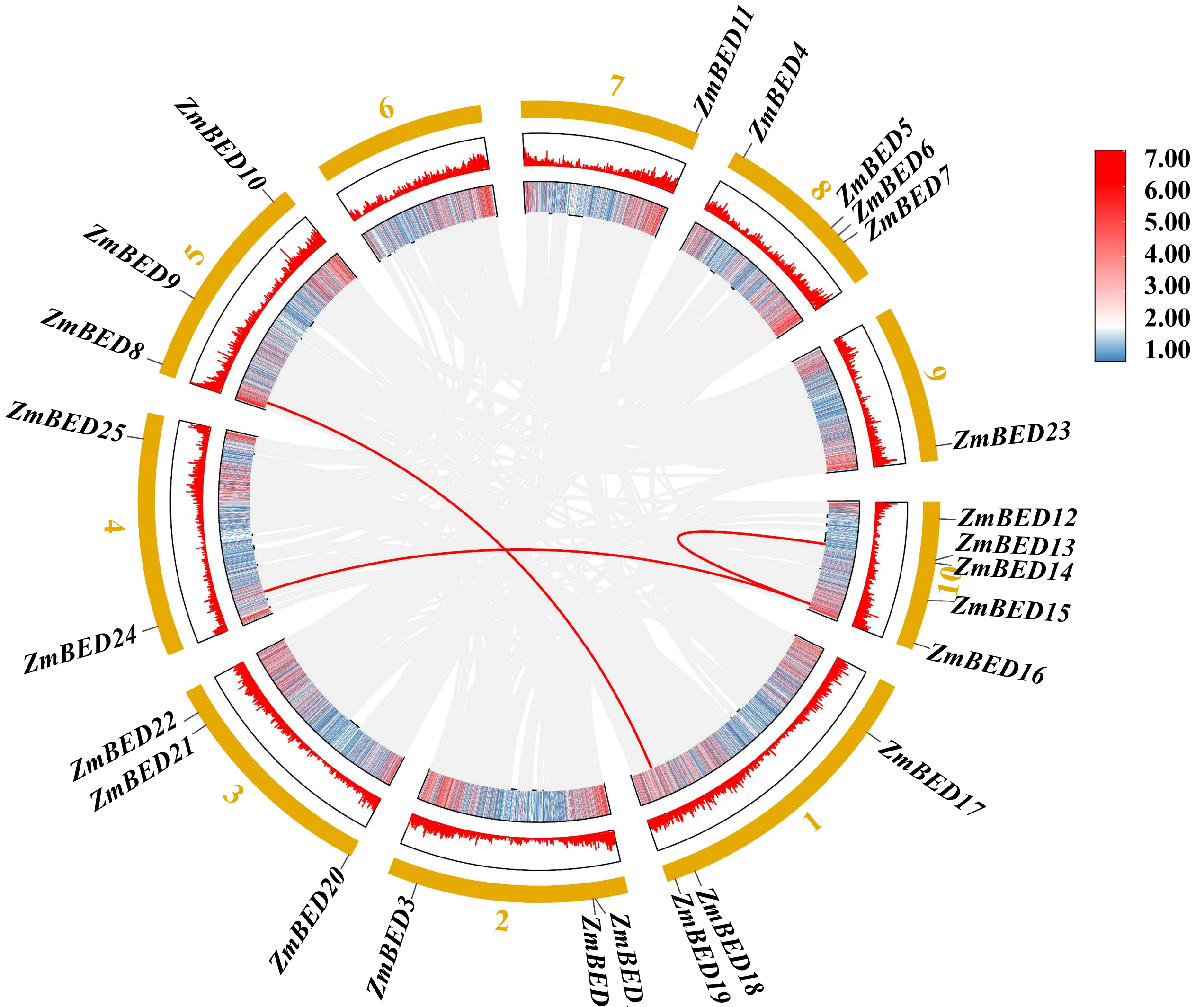

Supplementary Figure 3 Intraspecies synteny analysis of *ZmBED* members in maize.
Syntenic relationships among *ZmBED* genes are shown on maize chromosomes 1, 4, 5, and 10, involving five genes. Each syntenic connection is represented by a line.

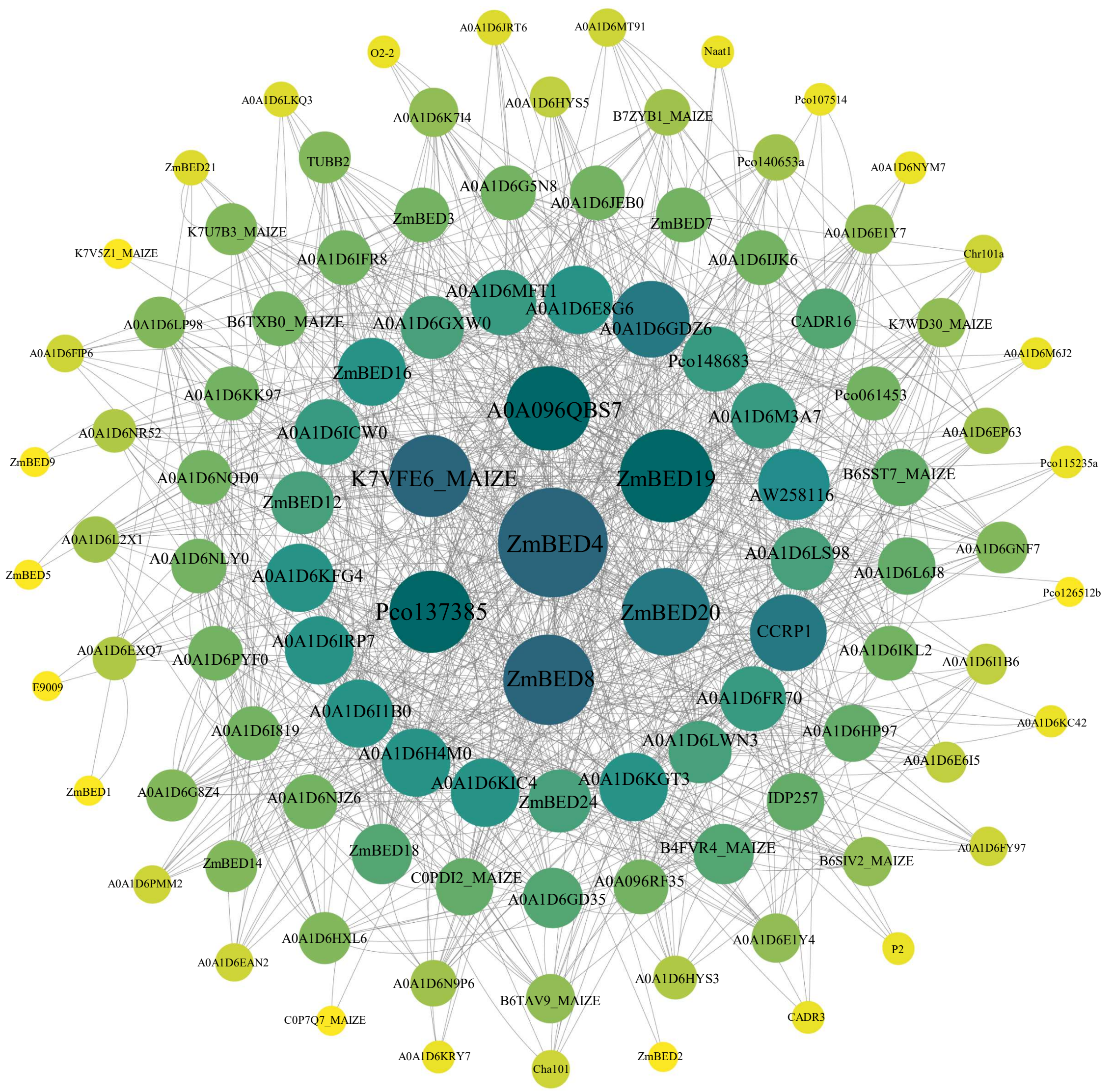

Supplementary Figure 4 Predicted protein-protein interaction network of ZmBED members.
Interactions were predicted using the STRING database (confidence threshold ≥ 0.700, where applicable). The network includes 90 interacting proteins (gray nodes) and 16 ZmBED members (colored nodes; colors as indicated in the figure). ZmBED4 (highlighted, e.g., in red) is the node with the highest degree (60 predicted interactors). Proteins A0A096QBS7 and CCR1 are the most highly connected non-ZmBED nodes. Edges represent predicted interactions.

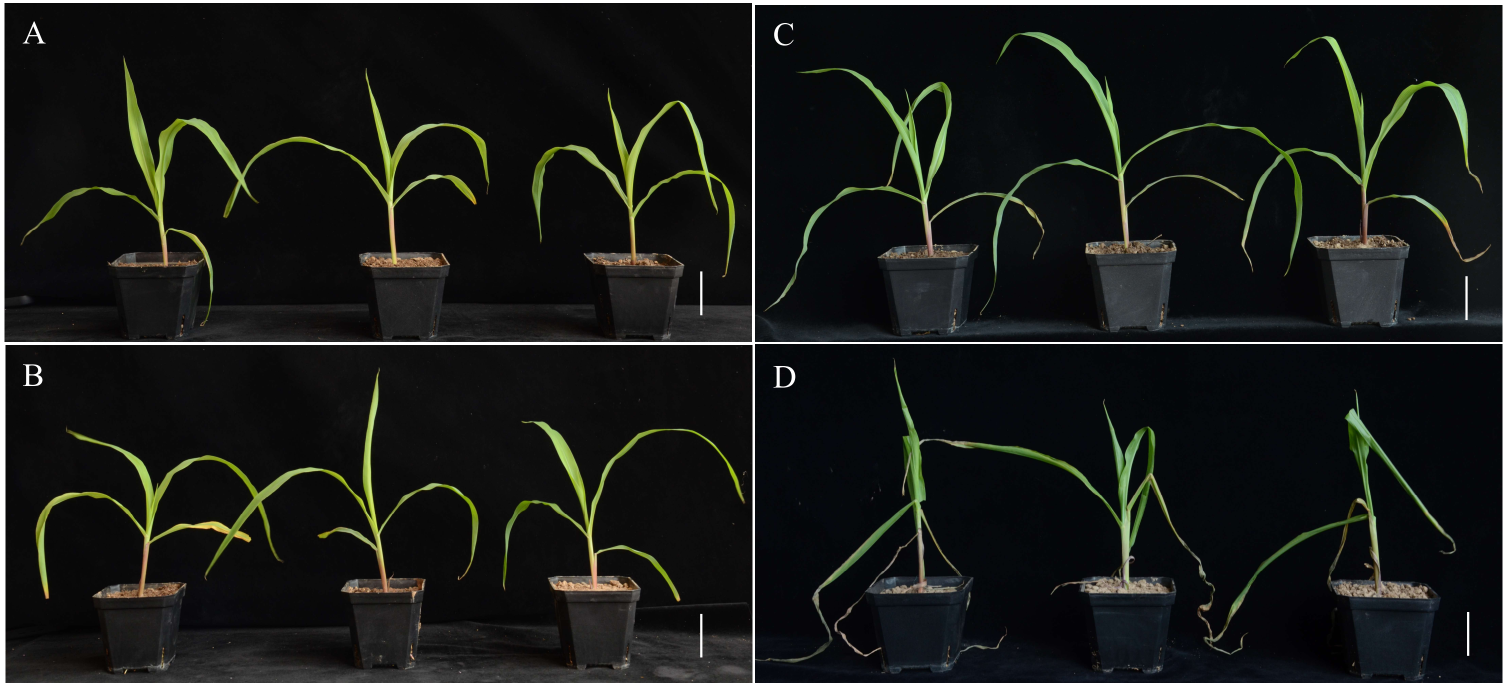

Supplementary Figure 5 Phenotypic response of maize (B73) to moderate drought stress.
(A, B) Representative plants before treatment (Day 0) showing no visible differences between control and drought groups; both exhibited expanded, dark-green leaves and uniform growth status. (C) Control plants after 14 days of well-watered conditions, displaying normal growth with erect leaves and typical green coloration. (D) Drought-treated plants after 14 days of exposure to 50% relative soil water content, showing stress-induced phenotypes including leaf curling, drooping, chlorotic leaf tips, and overall growth inhibition.

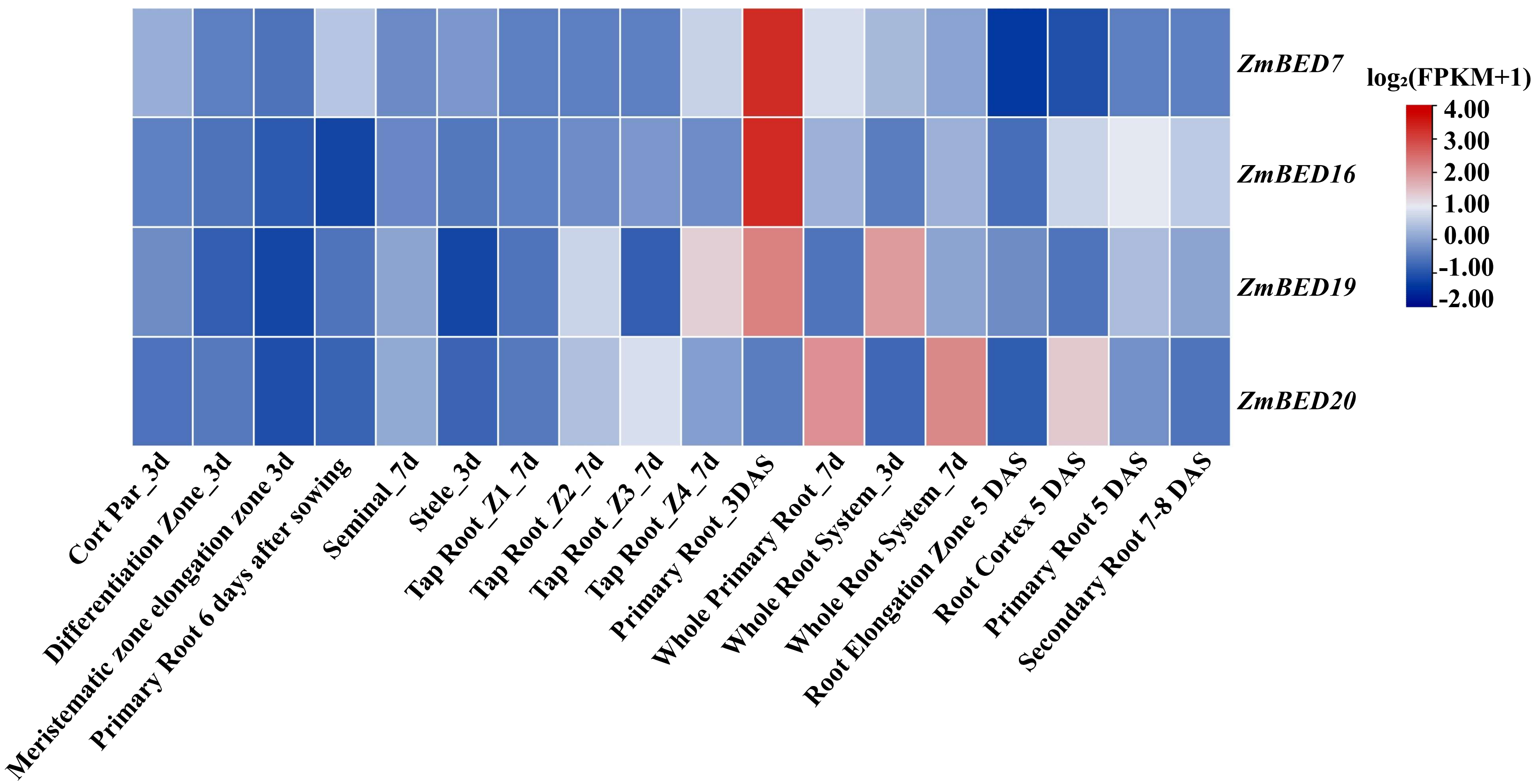

Supplementary Figure 6 Tissue-specific expression of *ZmBED* genes in maize roots at different developmental stages.
Expression data were obtained from the qTeller transcriptome database (maizeGDB). The heatmap displays expression levels across root tissues at different developmental stages. The color scale represents normalized expression values ($\log_2$(FPKM+1)), with red indicating high expression and blue indicating low expression.
